# Natural Diversity in Total Phenol, Flavonoids, Antioxidant Properties, and Essential Oil Composition of Iranian Populations of *Myrtus communis* L.

**DOI:** 10.3390/plants13243458

**Published:** 2024-12-10

**Authors:** Reza Yarahmadi, Hasan Mumivand, Abdollah Ehtesham Nia, Mohamad Reza Raji, Sergio Argento

**Affiliations:** 1 Department of Horticultural Sciences, Faculty of Agriculture, Lorestan University, Khorramabad P.O. Box 465, Iran; reza.yarahmadi1398@gmail.com (R.Y.); ab.ehteshamnia@gmail.com (A.E.N.); raji.m@lu.ac.ir (M.R.R.); 2 National Research Council of Italy, Institute of Biomolecular Chemistry (CNR-ICB), Via P. Gaifami 18, 95126 Catania, Italy; sergio.argento@cnr.it

**Keywords:** myrtle, 1,8-cineole, chemotype, phytochemical diversity

## Abstract

*Myrtus communis* L. (Myrtaceae), widely valued for its aromatic leaves and essential oil, plays a significant role in traditional medicine and modern phytotherapy. The variability in its essential oil composition and bioactive compounds across different populations underscores its potential for novel therapeutic discoveries and agricultural utilization. This study aimed to evaluate the phytochemical diversity of 12 selected Iranian *M. communis* populations in their natural habitats. Leaf samples were collected in 2023 from these native habitats to assess various parameters, including phenolic compounds (total phenols and total flavonoids), antioxidant capacity, essential oil content, and essential oil composition. The results indicated significant variations in phenolic content and antioxidant capacity across the populations. The Khoraman population, used as a control, exhibited the highest levels of total phenols and flavonoids, followed by the Sar-sarab and Yazd populations, while the Poldokhtar and Kermanshah populations showed the lowest levels. Additionally, the Poldokhtar and Hormozgan populations demonstrated the highest antioxidant capacities. Essential oil content ranged from 0.480 to 1.478%, with the Khoraman and Padeghan populations having the highest percentages of 1.631 and 1.478%, respectively. GC/MS analysis identified 29 distinct compounds in the essential oils, with major components including 1,8-cineole (22.34 to 45.66%), *α*-pinene (19.25 to 35.96%), linalool (7.79 to 18.76%), and *α*-terpineol (5.26 to 9.17%). The myrtle populations were categorized into four groups: (1) Khoraman; (2) Shiraz and Yazd; (3) Ilam, Sar-sarab, Poldokhtar, and Padeghan; and (4) Khuzestan, Kerman, Kermanshah, Kohgiluyeh–Boyer–Ahmad, and Hormozgan. Principal Component Analysis (PCA) corroborated the cluster analysis results, as populations within each group displayed similar distributions in the biplot.

## 1. Introduction

The genus *Myrtus* includes flowering plants, with approximately 16 species reported in areas of the Middle East and Asia [1]. *Myrtus communis* L., commonly known as the myrtle plant, is a fragrant evergreen shrub belonging to the Myrtaceae family. This shrub features numerous branching stems that can grow up to 5 m tall. As a diploid species (2n = 2x = 22) that is self-pollinating, myrtle grows in Mediterranean and Middle Eastern regions [2]. While it is cultivated in some parts of Iran, it predominantly grows wild across the Zagros Mountain range, at an altitude of 900 to 1700 m, with annual rainfall ranging from 400 to 600 mm [2,3]. Myrtle leaves are 3–5 cm long and contain tannins, flavonoids and volatile oils [1]. The plant is aromatic due to essential oil glands found in its leaves, fruits, and flowers [4]. Historically, *M. communis* has been valued for its anti-inflammatory, antiviral, antiseptic, and disinfectant properties. The medicinal value of the plant lies in its leaves, which contain 1.5–2% essential oil, comprising terpenes, sesquiterpenes, alcohols, esters, aldehydes, phenols, and ethers [5,6,7]. The major constituents of the essential oil include terpinen-4-ol, 1,8-cineole, linalool, and linalyl acetate [1,4,7]. Myrtle’s therapeutic properties include its use in topical treatments for cold sores, nasal mucosa inflammation, and as an antiseptic. It also has decongestant, astringent, tonic, and antiparasitic properties. Topical application is effective for treating wounds, acne, hemorrhoids, gum infections, and psoriasis. Additionally, it is used orally for respiratory tract disorders, sinusitis, dry coughs, and disorders of the digestive and urinary tracts [5,6,8]. Although metabolic processes in living organisms are supported by a deep evolutionary history and high stability, they can be influenced by various environmental factors such as soil characteristics, precipitation, light, temperature, and altitude [6,8,9]. When environmental conditions change, plants must adapt through biochemical pathways and processes [3,10]. Consequently, medicinal plant populations growing in different ecological conditions can generate a wide range of active substances, leading to variations in both the quantity and quality of these compounds [1,7]. This variability can, in turn, impact the therapeutic and biological activities of the plant [11]. Given that wild populations of medicinal plants are often diverse in both morphology and chemistry, any strategy for their use or integration into industry—whether by harvesting raw materials from natural habitats or by establishing domestication and breeding programs—must ensure the safety, stability, and efficacy of the materials. Consequently, it is crucial to thoroughly assess and identify the phytochemical properties and yield attributes of the plant germplasm [12,13]. When introducing a medicinal species into agricultural systems, particularly due to its economic value or threats to its wild populations, it is essential to prioritize a comprehensive strategy of domestication. This strategy should involve a detailed assessment of the species’ chemical, genetic, and eco-physiological aspects, as well as its production potential [14].

Numerous studies have investigated the essential oil profile in myrtle from various countries, including Tunisia [15], Iran [10,16], Turkey [17], Italy [18,19,20], and Greece [21]. These investigations have revealed significant variations in the phytochemical profiles of myrtle. Commonly identified main compounds in the essential oil include 1,8-cineole, *α*-pinene, limonene, and linalool [22]. Studies have also demonstrated that the ratio of *α*-pinene to 1,8-cineole varies significantly based on the geographic origin of the plant populations [3,23]. The composition of essential oil is thus strongly influenced by the plant’s collection location. Consequently, exploring the phytochemical diversity of natural populations across different regions is essential for identifying and introducing germplasm with high essential oil content and desirable key compounds for application in the food and pharmaceutical industries [23]. Loss of genetic diversity diminishes the ability of populations to withstand biological and abiotic stresses in their natural habitats, heightening the risk of genetic erosion and species extinction [19]. As such, the declining number and range of myrtle populations, coupled with excessive harvesting of wild specimens, highlight the need for domestication programs that select optimal populations for use in the pharmaceutical and cosmetic industries [19]. Organizing a conservation program to preserve myrtle’s natural genetic diversity and facilitate plant domestication can help prevent further genetic loss [22] and ensure the maintenance of high genetic diversity, particularly in environments facing intense anthropogenic pressures and potential climatic or environmental changes [2]. Information on genetic distance, along with the phytochemical profiles and morphological characteristics of plant populations, can aid breeders in improving breeding and domestication programs [1,2]. Consequently, assessing phytochemical diversity and implementing conservation programs are crucial for preserving myrtle germplasm [19]. Although numerous studies have examined the essential oil composition of myrtle populations in Iran, these investigations are often confined to specific regions or limited geographic areas. Additionally, there is a lack of comprehensive research that explores the essential oil composition, total phenols, flavonoids, and antioxidant activity across a broad range of populations from different geographic regions. The aim of the present study, therefore, was to assess the phytochemical diversity of wild myrtle populations in Iran, with a focus on the plant’s primary distribution areas. Specifically, we investigated total phenols, flavonoids, antioxidant activity, as well as the essential oil content and composition. This study seeks to identify the most promising populations for potential use in the pharmaceutical industry as well as for breeding and domestication efforts.

## 2. Results and Discussion

### 2.1. Total Phenols, Flavonoids, and Antioxidant Capacity

The results of mean comparisons showed that the Khoraman population, used as the control, and the Sar-sarab population had the highest levels of total phenols (22.62 ± 2.96 and 20.25 ± 8.04 mg GAE/g DW, respectively) and total flavonoids (10.22 ± 0.51 and 9.24 ± 0.14 mg RE/g DW, respectively). The Yazd population also exhibited high total phenol content. In contrast, the Kermanshah population had the lowest levels of both total phenols and total flavonoids (10.1 ± 5.00 mg GAE/g DW and 4.16 ± 0.26 mg RE/g DW, respectively). The Ilam and Shiraz populations also had relatively low levels of flavonoids (Table 1).

Numerous studies have demonstrated that myrtle extracts and essential oils possess significant antioxidant properties [11,16]. Research indicates that the antioxidant properties of plant extracts are strongly correlated with their phenolic levels [16,24]. High concentrations of phenolic compounds facilitate the transfer of hydrogen to free radicals, thereby enhancing the extract’s ability to inhibit oxidative processes [25]. This underscores the importance of phenolic compounds in understanding their antioxidant significance [26]. Additionally, studies have identified a positive relationship between the principal compounds in essential oils and their antioxidant capacity. For instance, 1,8-cineole has been linked to antioxidant activity in both DPPH and FRAP assays [16]. Increased activity of phenylalanine ammonia-lyase (PAL) during vegetative and flowering stages may contribute to the higher production of phenolic compounds. Furthermore, transcription factors and regulatory proteins have been implicated in the accumulation of phenolic compounds in plant tissues [27]. In this study, correlation analysis revealed a negative correlation between essential oil content and the antioxidant properties of myrtle. Conversely, a positive correlation was observed between total phenolic content and antioxidant activity. Notably, the correlation between total flavonoid content and antioxidant properties was particularly strong (Table S1). Populations such as Khoraman, Sar-sarab, Hormozgan, Poldokhtar, and Kerman, which exhibit the highest levels of phenols and flavonoids, also show the greatest antioxidant activity. These populations may have significant economic potential.

The biosynthesis and accumulation of secondary metabolites in plants are influenced by geographic origin of the species as well as biotic and abiotic factors [28,29]. Previous research indicates that factors such as light intensity, UV radiation (especially due to increased solar radiation at higher altitudes), temperature, altitude, and soil moisture significantly affect total phenolic content and antioxidant capacity [30,31,32,33]. In this study, we observed that geographical conditions, including latitude, longitude, and altitude, contribute to variations in phenolic compounds and antioxidant capacity among myrtle populations. Spitaler et al. [34] demonstrated that variations in phenolic compounds production among genetically similar populations along elevation gradients play a crucial role in inhibiting reactive oxygen species, which are likely generated by increased UV-B light [35]. Gene expression patterns related to the phenylpropanoid pathway, such as those encoding PAL and cinnamate-4-hydroxylase (C4H), are influenced by physiological and environmental conditions, which may account for variability in phenolic compounds [28,36]. Plants from cold climates, higher elevations, or semi-arid regions generally exhibit higher phenolic content [30,37]. Our findings also support this, showing that myrtle plants at higher elevations have higher phenolic contents. However, a plant’s response to its environment is largely influenced by its genetic factors [38]. The impact of drought stress on secondary metabolites in medicinal plants has been well-documented. For example, drought stress (low annual rainfall) often results in increased levels of flavonoids and phenolics [39]. This increase is attributed to the enhanced expression and activity of key enzymes involved in their biosynthesis, such as PAL, C4H, 4-coumarate-CoA ligase, and chalcone synthase [35,40].

### 2.2. Essential Oil

The essential oil content varied between 0.508 to 1.478% among the different *M. communis* populations. The Khoraman population, with 1.478%, and the Padeghan population, with 1.613%, had the highest essential oil content. In contrast, the populations from Shiraz, Hormozgan, and Yazd showed the lowest essential oil percentages, measuring 0.568, 0.508, and 0.628%, respectively (Table 1). Ghafouri and Rahimmalek [2] similarly reported that the essential oil content of myrtle leaves ranged from 1.06% in the Armand population of Chaharmahal-Bakhtiari province to 2.28% in the Darai population of Lorestan province. Other studies have documented a comparable range of 0.7 to 1.5% for Iranian *M. communis* populations [3,10,16,22]. In comparison, myrtle leaves from Italian populations contained essential oil percentages between 0.32 and 0.52% [18,41,42], from Tunisian populations between 0.44 and 0.6% [15,43], and from Montenegrin populations between 0.71% and 0.81% [44]. Overall, the essential oil content of the Iranian populations studied is significantly higher than that of European and North African varieties [2].

In this study, GC-FID and GC-MS analyses identified 29 different components in the essential oils from the various populations. The predominant components across all populations were 1,8-cineole (ranging from 22.34 to 45.66%), α-pinene (ranging from 19.25 to 35.96%), linalool (ranging from 7.79 to 18.76%), and α-terpineol (ranging from 5.26 to 9.17%). Notable quantities of geraniol (ranging from 1.03 to 2.67%), linalyl acetate (ranging from 1.17 to 6.41%), α-terpineol acetate (ranging from 2.12 to 3.60%), methyl eugenol (ranging from 0.54 to 3.24%), and caryophyllene (ranging from 0.20 to 2.07%) were also observed in the essential oils of most populations (Table 2). Significant variations were found between *M. communis* populations in the percentage of essential oil constituents. For instance, the level of 1,8-cineole in the essential oils of the Ilam and Sar-sarab populations was 2.03 times higher than that in the Khoraman population. The highest level of 1,8-cineole was noted in the Ilam, Sar-sarab, Kermanshah, and Poldokhtar populations (45.66, 45.39, 41.73, and 41.48%, respectively). The highest level of α-pinene (34.96%) was found in the Khuzestan population, while the Shiraz and Sar-sarab populations exhibited the lowest levels of α-pinene (19.58 and 19.25%, respectively). The highest and lowest levels of linalool were recorded in the Shiraz (18.76%) and Hormozgan (7.59%) populations, respectively. Additionally, the Khoraman and Sar-sarab populations showed the highest levels of α-terpineol (9.17 and 8.61%, respectively), whereas the Kermanshah population had the lowest level (5.26%) (Table 2).

Numerous studies have documented the diversity of essential oils in *M. communis* from various regions, including Iran [3,10,16,22,23], Italy [18,20,40,41], Tunisia [15], and Algeria [45]. These studies have consistently identified α-pinene, limonene, 1,8-cineole, α-terpineol, linalool, and linalool acetate as the primary components of *M. communis* essential oil. Rahimmalek et al. [23] assessed the essential oil components of 21 myrtle populations in southwestern Iran and found significant phytochemical polymorphism among these populations. The primary constituents of the essential oils identified were α-pinene, 1,8-cineole, limonene, linalool, α-terpineol, and linalool acetate. They also distinguished two chemotypes: α-pinene/1,8-cineole and limonene/α-pinene. Bajalan and Ghasemi Pirbalouti [22] reported that the main constituents of the *M. communis* essential oil were α-pinene (24.42% to 31.57%), limonene (trace to 23.41%), 1,8-cineole (5.92% to 21.21%), linalool (8.72% to 11.56%), α-terpineol (7.04% to 8.12%), geranyl acetate (2.33% to 5.12%), and linalyl acetate (trace to 7.12%). Among these, α-pinene was the dominant component across all samples, followed by limonene and 1,8-cineole. In the current study, the primary constituents of the essential oils were identified as 1,8-cineole (22.34% to 45.66%), followed by α-pinene (19.25% to 34.96%) and linalool (9.16% to 18.76%). However, the Khuzestan population was noted for having α-pinene as the most abundant component of its essential oil.

The quantity and quality of active compounds in medicinal plants are primarily determined by genetic factors. However, environmental factors such as temperature, altitude, light intensity, and humidity also play a significant role in shaping these traits [22,46]. Although there is considerable diversity in the phytochemical composition of the *M. communis* populations studied in this research, populations located geographically closer to each other displayed similar compositions, suggesting that climatic conditions may influence genetic factors. Among the various environmental and geographical factors, altitude is one of the most influential in affecting plant growth and biochemical properties [47]. In high-altitude areas, increased solar radiation, enhanced light quality, and a greater day–night temperature differential generally promote photosynthesis and reduce nighttime respiration. This, in turn, leads to higher carbohydrate reserves, which act as a protective mechanism against cold stress in lower temperatures. Moreover, increased photosynthesis and carbohydrate accumulation are crucial to providing the carbon skeleton needed to initiate the biosynthesis of secondary metabolites [36,48]. For instance, in *Thymus fallax*, studies have shown that as altitude increases, the concentrations of thymol and carvacrol in the essential oil also rise significantly [49].

In the current study, *M. communis* populations are categorized into four distinct chemotypes. Chemotype 1,8-cineole includes the Sar-sarab, Poldokhtar, Ilam, and Padeghan populations, which are characterized by similar latitudinal and longitudinal coordinates. Chemotype α-pinene/1,8-cineole is represented solely by the Khuzestan population. This unique chemotype is likely attributed to the area’s high relative humidity, relatively low elevation, and the alluvial conditions of the Khuzestan plain, setting it apart from other populations. Chemotype 1,8-cineole/α-pinene includes the Kermanshah, Hormozgan, Khoraman, and Kohgiluyeh–Boyer–Ahmad populations, which are located within relatively close latitudinal ranges. Despite its differing latitude, the Kerman population is included in this group due to its higher altitude, which correlates with lower temperatures. Chemotype 1,8-cineole/α-pinene/linalool comprises the Yazd and Shiraz populations, which share similar latitudinal and longitudinal coordinates as well as elevation, indicating comparable climatic conditions. Rahimmalek et al. [23] found that key essential oil components, including α-pinene, 1,8-cineole, limonene, linalool, and α-terpineol, exhibited a strong correlation with environmental factors such as soil and climatic conditions at the collection sites. Similar findings were observed in *M. communis* essential oil from specific regions in Italy [18], Montenegro [44], and Tunisia [15], highlighting the link between environmental factors and chemotypes. Shahbazian et al. [10] identified significant variation in essential oil components among 14 *M. communis* populations in Fars Province, Iran, documenting 23 chemical compounds in the essential oil. Dominant components included α-pinene (2.35 to 53.09%), linalool acetate (0 to 45.3%), caryophyllene oxide (0.97 to 21.8%), germacrene-D (0.19 to 19%), α-humulene (0 to 18.97%), 1,8-cineole (0 to 18%), limonene (0 to 17.4%), and p-cymene (0 to 13.2%).

Ghasemi Pirbalouti and Craker [10] reported that essential oils from Myrtle populations in southwestern Iran (Khuzestan and Lorestan provinces) contained high levels of oxygenated monoterpenes (24.7 to 66.9%), such as 1,8-cineole, *α*-terpineol, and linalool, as well as monoterpene hydrocarbons (22.3 to 58.5%), such as *α*-terpinene and limonene. Studies on *M. communis* essential oils from Tunisia [50] and Italy [18,51] identified 15 terpenoid compounds, with major terpenes including *α*-terpinene, myrtenyl acetate, 1,8-cineole, *α*-limonene, and linalyl acetate. Minor compounds found included β-ocimene, 3-carene, terpinene, *p*-cymene, *β*-caryophyllene, terpinolene, and humulene. These findings suggest that terpenoid composition is strongly influenced by genetic factors, as supported by another research [23,50]. Overall, the bioactive compounds in medicinal plants are primarily governed by genetic factors but are also significantly affected by environmental conditions such as altitude, slope, latitude, temperature, light, and relative humidity. These factors influence plant growth and the quantity and quality of bioactive compounds, including alkaloids, glycosides, steroids, and essential oils [29,52,53].

### 2.3. Cluster Analysis

**Figure 1:**
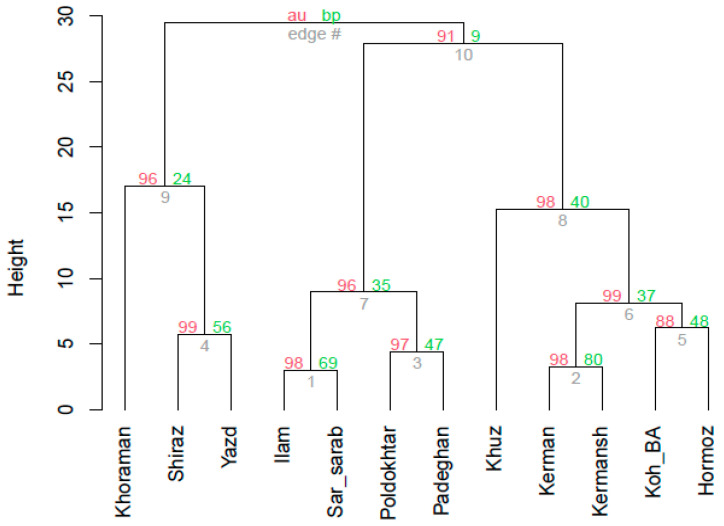
Cluster analysis of Iranian myrtle populations according to their essential oil constituents using Ward’s minimum variance; au: approximately unbiased value; clusters with au ≥ 95% are considered to be strongly supported by data; bp: bootstrap probability value shows the frequency that the cluster is identified in bootstrap copies.

Based on the cluster analysis of essential oil compositions, the studied *M. communis* populations were categorized into four main groups (Figure 1). The first group included the Khoraman population, which exhibited the lowest levels of 1,8-cineole but the highest concentrations of α-terpineol, linalyl acetate, and α-terpineol acetate among all populations. This group also had a relatively high linalool content. The second group consisted of the Shiraz and Yazd populations, which had the highest linalool levels among the studied populations, although their levels of 1,8-cineole and α-pinene were moderate. The third group included the Ilam, Sar-sarab, Poldokhtar, and Padeghan populations, characterized by their notably high levels of 1,8-cineole. The fourth group comprised the Khuzestan, Kerman, Kermanshah, Kohgiluyeh–Boyer–Ahmad, and Hormozgan populations, which displayed the highest percentages of α-pinene (Table 2). High approximately unbiased (AU) values at the boundaries of each group indicate strong support for the cluster analysis, suggesting that the dendrogram is robust and well-supported by the data. The Bootstrap Probability (BP) value indicates the frequency with which the cluster appears in bootstrap samples. Previous studies have also assessed the essential oil compositions of various *M. communis* populations in specific geographic regions of Iran [3,10,23]. Alongside genetic factors, ecological conditions and geographical distance play crucial roles in the evolution and distribution of plant species across different regions. For wild populations, the geographical distance and gene flow between populations are key determinants of genetic distance [54]. The findings of this study further revealed that populations located in close proximity to one another geographically were clustered together. The classification of 14 myrtle populations from southern Iran (Fars Province), based on the main components of their essential oils, utilizing hierarchical cluster analysis (HCA) and principal component analysis (PCA), resulted in the populations being grouped into four separate clusters. These results suggest that genetic factors have a more significant influence than ecological conditions in driving the variation observed in the essential oils [55]. Ghasemi Pirbalouti and Craker [3] classified 12 *M. communis* populations into four distinct groups based on their essential oil compositions. The first group, comprising populations from Madan–Chaharmahal–Bakhtiari, Dehdasht–Kohgiluyeh–Boyer–Ahmad, Cheshmaeh Ali–Fars, and Seyedan–Fars, was characterized by high levels of α-pinene, 1,8-cineole, linalool, and limonene, with the highest linalool content observed among all populations. The second group, including the Seyedan-Fars population, also had elevated levels of α-pinene, linalool, 1,8-cineole, and limonene. The third group consisted of three populations Chavoni–Andimeshk, Mongere–Andimeshk, and Sardasht–Dezful, which had essential oils with the highest concentrations of α-pinene and 1,8-cineole compared to other groups. The fourth group included three populations—Dinarvand–Lorestan, Tangehaft–Lorestan, and Pelazh Sad Dez–Khuzestan—which were noted for their essential oils containing α-pinene, 1,8-cineole, and limonene, with this group having the highest limonene content among the groups. While genetic differences primarily account for the variations and similarities in essential oil compositions among populations, these differences may also be influenced by enzymatic reactions resulting from water scarcity or other environmental stresses [3]. Hendawy and Khalid [56] reported that environmental factors affecting enzyme activity and metabolic pathways can alter essential oil content and composition. A significant negative correlation was noted between oxygenated monoterpenes and hydrocarbon monoterpenes, which may be explained by changes in the biosynthetic pathways for these compounds [3].

### 2.4. Principal Component Analysis (PCA)

**Figure 2:**
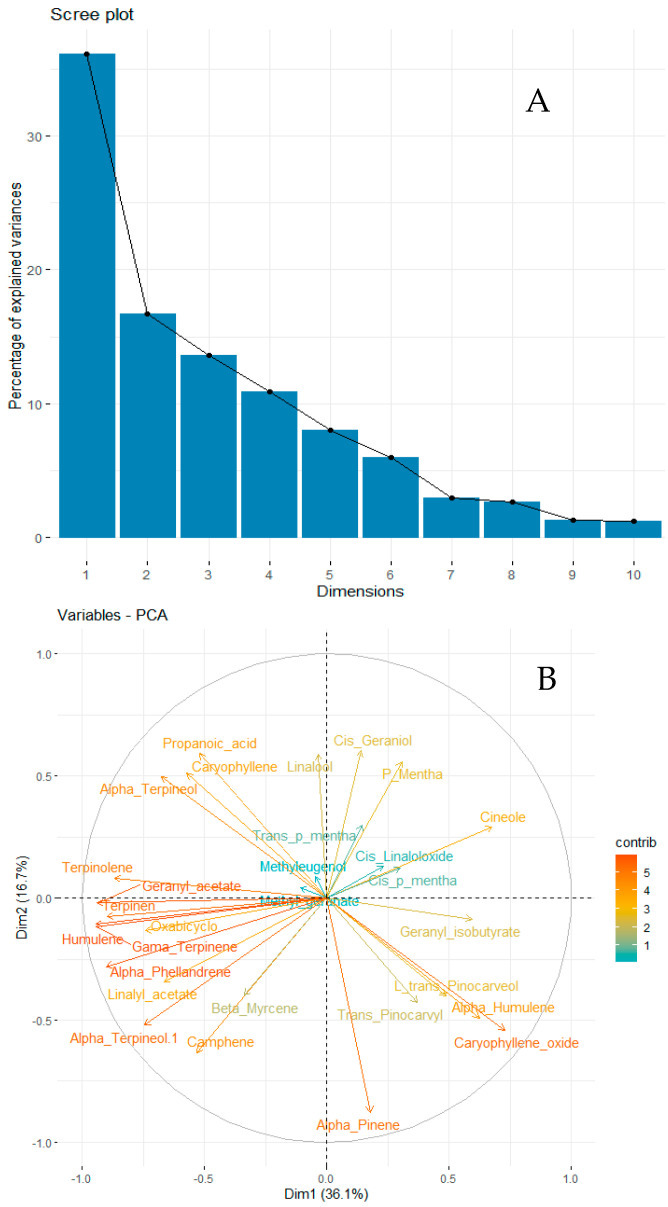
(**A**) The 10 principal components and their percentage of variance are explained. The first 4 principal components were utilized to generate the biplot for the principal component analysis. (**B**) The contribution of each essential oil constituent is depicted in two dimensions using gradient scales and color intensity. Vectors farther from the plot center represent higher contribution values. Narrow angles between vectors indicate similarity, while wide angles suggest negative correlation.

**Figure 3:**
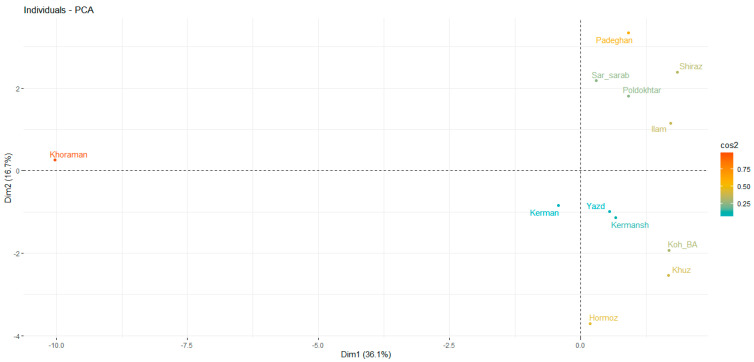
Biplot from principal component analysis of essential oil constituents in Iranian myrtle populations.

To determine whether there is any ordination among the studied essential oils, a PCA was conducted using the percentages of the essential oil constituents. Eigenvalues were analyzed to determine the components that influence the grouping of populations. The first principal component accounted for over 31% of the total variance, and together, the first four components explained more than 70% of the variance (Figure 2A). Consequently, the remaining principal components, which contribute less significantly, can be disregarded. Since the principal components are uncorrelated, each component reveals different aspects of the data that are not captured by the others. To differentiate the populations and their essential oil compositions, a two-dimensional plot was generated using the first four principal components. This PCA biplot was constructed to visualize the data. The importance of each compound in the PCA was evaluated using the Contribution index, with vectors further from the plot center indicating higher contributions. Compounds located in the same group with vector angles less than 90 degrees from each other show a direct relationship and positive correlation. An angle of less than 90 degrees in the PCA biplot approximates the correlation coefficient, aligning with Pearson correlation results between traits. For instance, the compounds 1,8-cineole, (Z)-geraniol, and (Z)-p-mentha-6,8-dien-2-ol were most influential in the first component. The second component was primarily driven by linalool, α-terpineol, α-terpineol acetate, caryophyllene, and propanoic acid, 2-methyl-, 2-methylpropyl ester. α-Phellandrene, γ-terpinene, linalyl acetate, camphene, α-terpineol, and humulene contributed significantly to the third component. Caryophyllene oxide, α-humulene epoxide, α-pinene, and geranyl isobutyrate were most prominent in the fourth component (Figure 2B). Figure 3 illustrates the PCA biplot based on these components. The plot shows the studied populations grouped into distinct clusters that reflect their essential oil composition averages. The significance of each population’s contribution to the PCA was assessed using the cos^2^ index, with the Khoraman population having the most notable impact on the plot. According to the biplot, the populations from Kerman, Yazd, and Kermanshah clustered together. The second axis revealed distinct population groups, with Khoraman, Padeghan, and Hormozgan showing the greatest genetic distances and cos^2^ values. The Sar-sarab and Poldokhtar populations also formed a cluster. Additionally, the populations from Kohgiluyeh–Boyer–Ahmad and Khuzestan were identified as a separate group (Figure 3).

## 3. Materials and Methods

### 3.1. Plant Materials

**Figure 4:**
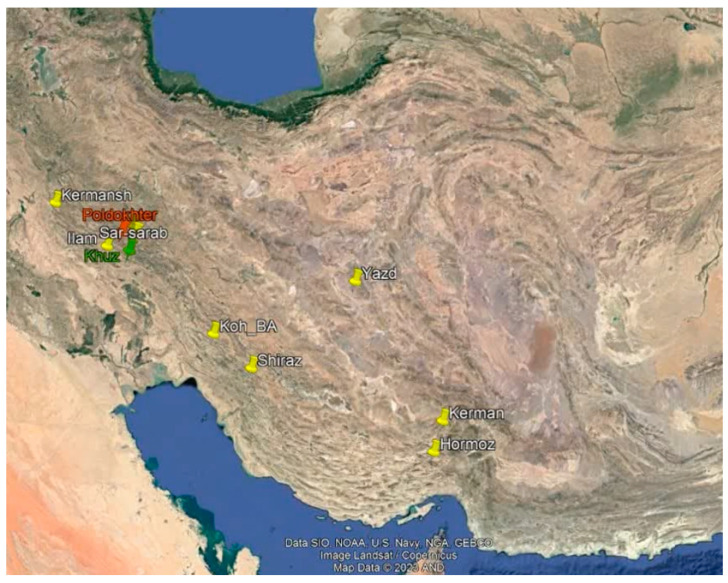
Map illustrating the distribution of myrtle populations collected across Iran.

To assess the phytochemical diversity among various myrtle populations, 11 natural habitats with suitable distribution across Iran were selected. These populations were from the western, southwestern, southern, and central regions, encompassing areas with the highest myrtle distribution in the country. The selected habitats were located in the provinces of Lorestan (two regions), Khuzestan, Kermanshah, Ilam, Kohgiluyeh–Boyer–Ahmad, Yazd, Kerman, Hormozgan, and Fars (Figure 4) (Table 3). Additionally, one population was sourced from Khoraman Pharmaceutical Co. as a cultivated genotype for comparison. During natural habitat visits in June and July 2022, leaf samples were collected from each population at the full flowering stage. Plant identification was conducted by biologist using the Flora of Iran. Herbarium specimens were also prepared and assigned a voucher number of LU-1005 by the Herbarium of the Faculty of Natural Resources, University of Lorestan. The leaf samples (2 kg) from the myrtle populations were transported to the Horticultural Sciences Laboratory at Lorestan University and dried in the shade.

### 3.2. Extraction

Ten grams of dried and ground plant material were combined with 100 mL of an 80% methanol solution. The mixture was then shaken at room temperature for 72 h. Afterward, the mixture was filtered through filter paper, and the methanol was removed from the extract using a rotary evaporator (Heidolph, Schwabach, Bavaria, Germany). The resulting pure extract was then stored in glass vials at 4 °C until further analysis [24].

### 3.3. Total Phenols

To determine the total phenolic content, 0.5 mL of 10% Folin–Ciocalteu reagent were combined with 4 mL of 1 M Na_2_CO_3_ solution. Next, 0.5 mL of each extract were introduced into this solution. The mixtures were left to stand at room temperature for 15 min. Absorbance was recorded at 765 nm using a Shimadzu UV-1700 spectrophotometer. All measurements were taken in triplicate. The total phenol content was calculated as mg gallic acid equivalent (GAE) per gram of dry weight (DW) using a standard curve of gallic acid [57].

### 3.4. Total Flavonoids

To measure the total flavonoid content, 0.1 mL of 10% aluminum chloride solution (10%) was first added to test tubes, followed by 0.1 mL of potassium acetate (1 M) and 2.8 mL of distilled water. Subsequently, 0.5 mL of the methanolic leaf extract were mixed into the solution. The samples were then kept in the dark for 30 min. Absorbance at 415 nm was recorded using a Shimadzu UV-1700 spectrophotometer. The total flavonoid content was calculated as mg rutin equivalent (RE) per gram of DW, based on a rutin standard curve [58].

### 3.5. Antioxidant Property

The antioxidant capacity of the extracts was assessed using the FRAP method, which measures the reduction of Fe^3+^-TPTZ to Fe^2+^-TPTZ. A fresh FRAP solution was made by combining acetate buffer, TPTZ, and iron chloride in a 10:1:1 ratio, and maintained at 37 °C. Then, 180 µL of this FRAP solution was combined with 20 µL of the methanolic extract and incubated at 37 °C for 8 min. The absorbance of the resulting solution was recorded at 593 nm using a Shimadzu UV-1700 spectrophotometer. The antioxidant capacity was reported as µmol Fe/g DW [59].

### 3.6. Essential Oil Isolation and Analysis

Essential oils were isolated via steam distillation with a Clevenger apparatus; 50 g of dried, finely ground leaves were heated in the Clevenger flask for 3 h. The resulting essential oil was then dehydrated using sodium sulfate and weighed precisely. The percentage of essential oil was determined based on the weight of the oil extracted from 100 g of plant material (*w*/*w*). The essential oil was kept in dark glass vials at 4 °C until further analysis [60]. For essential oil analysis, an Agilent gas chromatograph (Model SCION SQ W/436, SSL-T21) from Agilent Technologies (Santa Clara, CA, USA), coupled with a SCION-MS single Quadrupole-MS mass spectrometer was used. Essential oil compounds were separated using a DB-5MS column (Agilent). The ionization energy was set at 70 EV, and the ionization source temperature was maintained at 270 °C. The temperature of the oven was programmed to increase from 40 to 280 °C at a rate of 7 °C/min, then held at 280 °C for 5 min. Helium was used as the carrier gas, flowing at 1 mL/min. The injector and interface temperatures were maintained at 280 and 260 °C, respectively, with a split ratio of 1:50. For quantification of the essential oil constituents, gas chromatography with a flame ionization detector (GC-FID) Model SCION SQ W/436 SSL-T21 from Agilent Technologies, USA equipped with a DB-5 fused-silica capillary column was used. The analytical conditions for oven temperature, injector temperature, carrier gas, and split ratio were the same as those used for GC-MS. The identification of essential oil constituents was achieved (1) by determining their retention indices through temperature-programmed analysis with *n*-alkanes (C_8_–C_20_) on the DB-5 column and comparing retention indices with literature values (2) by comparing retention times to authentic standards and/or (3) matching mass spectra against internal libraries (NIST11) [61,62].

### 3.7. Data Analysis

Data analysis was performed using a completely randomized design with three replicates, and the variance analysis was conducted with SAS software version 9.2. The comparison of means was done using Duncan’s multiple range test. Cluster analysis of the myrtle populations was conducted using Euclidean distance and the Ward method within the pvclust package of R software (version 4.4.1.). Principal component analysis was also conducted using the factoextra package in R software.

## 4. Conclusions

The results of this study reveal substantial phytochemical diversity among Iranian *M. communis* populations, particularly in terms of phenolic compounds, antioxidant activity, as well as essential oil content and composition. Among the populations studied, Khoraman, Sar-sarab, and Yazd exhibited the highest levels of total phenols and flavonoids. The Khoraman and Hormozgan populations also showed the greatest antioxidant capacities. Additionally, Khoraman and Padeghan demonstrated the highest essential oil content. The essential oils of all studied populations predominantly contained 1,8-cineole, linalool, α-pinene, and α-terpineol. Four distinct chemotypes of Myrtle were identified. Chemotype 1,8-cineole included the Sar-sarab, Poldokhtar, Ilam, and Padeghan populations. Chemotype α-pinene/1,8-cineole was represented exclusively by the Khuzestan population. The Kermanshah, Hormozgan, Kerman, Khoraman, and Kohgiluyeh–Boyer–Ahmad populations were classified under the 1,8-cineole/α-pinene chemotype. The 1,8-cineole/α-pinene/linalool chemotype included the Yazd and Shiraz populations. Cluster analysis further divided the populations into four groups: (1) Khoraman; (2) Shiraz and Yazd; (3) Ilam, Sar-sarab, Poldokhtar, and Padeghan; and (4) Khuzestan, Kerman, Kermanshah, Kohgiluyeh–Boyer–Ahmad, and Hormozgan. Differences in essential oil contents between these populations may be influenced by geographical location, climatic conditions at the sampling sites, as well as genetic factors. This information is crucial for selecting optimal *M. communis* populations for domestication and cultivation, with potential applications in the pharmaceutical and food industries.

## Figures and Tables

**Table 1:** Total phenol and flavonoid contents, antioxidant activity, and essential oil content of the leaves of Iranian myrtle populations.

Population	Total Phenol (mg GAE/g DW)	Total Flavonoid (mg RE/g DW)	Antioxidant Activity (µg Fe/g DW)	Essential Oils Content (mg/100 g DW)
Khoraman	22.62 ± 2.96 a	10.22 ± 0.77 a	437 ± 0.51 a	1.47 ± 0.08 a
Sar-sarab	20.25 ± 8.04 abc	9.24 ± 0.14 ab	359 ± 3.17 d	0.77 ± 0.11 d
Padeghan	17.18 ± 9.68 cd	6.96 ± 0.77 cde	344 ± 2.63 e	1.16 ± 0.14 b
Poldakhter	14.80 ± 6.11 d	8.25 ± 0.54 bc	388 ± 1.04 c	0.93 ± 0.09 c
Ilam	19.29 ± 3.66 bc	5.84 ± 0.60 e	367 ± 0.84 d	0.81 ± 0.06 cd
Kermanshah	10.10 ± 5.00 e	4.16 ± 0.26 f	385 ± 0.99 c	0.76 ± 0.05 de
Koh_BA	18.21 ± 3.50 bc	8.07 ± 0.67 bc	327 ± 2.29 f	0.71 ± 0.07 de
Khuzestan	17.748 ± 10.87 cd	6.36 ± 1.17 de	392 ± 0.98 c	0.68 ± 0.11 def
Kerman	17.483 ± 12.29 cd	7.60 ± 1.05 cd	391 ± 0.52 c	0.81 ± 0.07 cd
Yazd	21.13 ± 6.92 ab	7.92 ± 0.44 bc	313 ± 1.69 g	0.62 ± 0.03 efg
Shiraz	15.03 ± 6.33 d	6.23 ± 1.23 e	306 ± 1.80 g	0.50 ± 0.10 g
Hormozgan	17.38 ± 5.46 cd	7.90 ± 0.63 c	410 ± 2.56 b	0.56 ± 0.01 fg

Means with similar letters in each column do not have a significant difference based on Duncan’s multiple range test.

**Table 2:** The essential oil constituents of the leaves of Iranian myrtle populations.

No	Constituents	RI ^a^	KI ^b^	Khoraman	Sar-sarab	Padeghan	Poldakhter	Ilam	Kermanshah	Koh_BA	Khuzestan	Kerman	Yazd	Shiraz	Hormozan
1	Propanoic acid, 2-methyl-, 2-methylpropyl ester	906	914.59	1.70 ± 0.08	1.02 ± 0.01	1.74 ± 0.04	0.55 ± 0.025	0.65 ± 0.03	0.26 ± 0.005	0.28 ± 0.02	0.74 ± 0.03	0.66 ± 0.005	1.10 ± 0.02	1.08 ± 0.04	0.35 ± 0.004
2	*α*-Pinene	931	940.13	21.28 ± 1.07	19.25 ± 0.79	19.54 ± 1.15	21.46 ± 1.19	20.38 ± 0.15	25.55 ± 1.04	27.56 ± 0.40	34.96 ± 0.80	24.43 ± 1.00	23.21 ± 0.80	19.58 ± 1.11	29.81 ± 1.68
3	Camphene	947	953.89	0.16 ± 0.01	0.12 ± 0.005	-	-	-	0.11 ± 0.01	0.10 ± 0.002	0.10 ± 0.01	0.13 ± 0.01	0.11 ± 0.01	-	0.11 ± 0.001
4	*β*-Myrcene	988	992.56	0.61 ± 0.03	0.45 ± 0.004	0.28 ± 0.02	0.18 ± 0.01	0.39 ± 0.04	0.59 ± 0.01	0.36 ± 0.03	0.29 ± 0.007	0.75 ± 0.03	0.88 ± 0.05	0.36 ± 0.01	0.57 ± 0.03
5	*α*-Phellandrene	1002	1005.37	1.33 ± 0.22	0.51 ± 0.07	0.48 ± 0.07	0.34 ± 0.01	0.34 ± 0.03	0.61 ± 0.11	0.49 ± 0.02	0.42 ± 0.02	0.84 ± 0.11	0.48 ± 0.03	0.30 ± 0.02	0.77 ± 0.11
6	1-8-Cineole	1031	1037.58	22.34 ± 0.08	45.39 ± 0.25	39.67 ± 1.53	41.48 ± 1.12	45.66 ± 1.39	41.73 ± 1.41	35.86 ± 0.93	31.24 ± 1.03	39.58 ± 0.74	33.40 ± 0.6	35.34 ± 0.90	38.27 ± 0.17
7	*γ*-Terpinene	1056	1058.57	0.77 ± 0.02	0.23 ± 0.01	0.25 ± 0.02	0.26 ± 0.01	0.20 ± 0.009	0.23 ± 0.005	0.20 ± 0.01	0.20 ± 0.001	0.27 ± 0.01	0.21 ± 0.01	0.21 ± 0.02	0.44 ± 0.008
8	(*Z*)-Linalool oxide	1066	1072.52	-	0.15 ± 0.01	0.17 ± 0.01	-	-	-	0.12 ± 0.004	-	0.15 ± 0.01	0.17 ± 0.02	0.09 ± 0.01	0.09 ± 0.01
9	Terpinolene	1083	1089.78	0.80 ± 0.09	0.22 ± 0.02	0.26 ± 0.01	0.21 ± 0.01	0.18 ± 0.02	0.23 ± 0.005	0.22 ± 0.01	0.23 ± 0.01	-	0.23 ± 0.01	0.24 ± 0.02	0.23 ± 0.01
10	Linalool	1101	1104.89	13.54 ± 1.17	11.31 ± 0.63	15.49 ± 0.78	12.32 ± 1.01	10.69 ± 0.96	9.16 ± 0.64	11.71 ± 0.64	10.84 ± 0.41	10.70 ± 0.07	16.57 ± 0.60	18.76 ± 1.11	7.59 ± 0.60
11	(*E*)-Pinocarveol	1139	1145.25	0.08 ± 0.02	0.59 ± 0.03	0.41 ± 0.03	0.21 ± 0.005	0.34 ± 0.04	0.35 ± 0.02	0.88 ± 0.06	0.45 ± 0.02	0.46 ± 0.02	0.45 ± 0.03	0.22 ± 0.01	0.52 ± 0.01
12	Terpinen-4-ol	1180	1184.74	0.78 ± 0.01	0.51 ± 0.02	0.48 ± 0.03	0.47 ± 0.02	0.41 ± 0.01	0.45 ± 0.01	0.40 ± 0.04	0.39 ± 0.02	0.54 ± 0.02	0.48 ± 0.02	0.44 ± 0.03	0.62 ± 0.01
13	(*E*)-*p*-mentha-1(7),8-dien-2-ol	1185	1192.83	-	0.20 ± 0.005	0.36 ± 0.41	-	0.10 ± 0.005	0.18 ± 0.01	-	-	0.20 ± 0.01	-	-	0.14 ± 0.01
14	*α*-Terpineol	1190	1201.53	9.17 ± 0.16	8.61 ± 0.63	7.18 ± 0.12	7.11 ± 0.33	6.60 ± 0.66	5.26 ± 0.02	6.38 ± 0.30	6.02 ± 0.20	6.39 ± 0.45	6.39 ± 0.25	6.86 ± 0.25	6.43 ± 0.11
15	(*Z*)-*p*-Mentha-6,8-dien-2-ol	1226	1222.87	-	0.25 ± 0.01	0.23 ± 0.006	-	0.11 ± 0.005	-	0.22 ± 0.007	-	0.17 ± 0.01	-	0.38 ± 0.02	-
16	(*Z*)-*p*-mentha-1(7),8-dien-2-ol	1231	1232.72	-	0.21 ± 0.01	0.23 ± 0.01	0.37 ± 0.03	0.26 ± 0.02	0.44 ± 0.005	-	0.21 ± 0.01	0.18 ± 0.008	0.51 ± 0.01	-	-
17	(*Z*)-Geraniol	1229	1240.75	1.51 ± 0.17	2.03 ± 0.09	2.20 ± 0.04	2.67 ± 0.05	1.86 ± 0.09	1.46 ± 0.01	1.31 ± 0.08	1.03 ± 0.08	1.65 ± 0.11	2.10 ± 0.0.3	1.85 ± 0.05	1.87 ± 0.09
18	Linalyl acetate	1252	1249.02	6.41 ± 0.11	1.17 ± 0.06	2.48 ± 0.37	2.81 ± 0.35	2.62 ± 0.06	4.39 ± 0.19	2.91 ± 0.11	2.26 ± 0.02	4.26 ± 0.34	5.38 ± 0.27	3.46 ± 0.06	4.27 ± 0.52
19	(*E*)-Pinocarvyl acetate	1301	1297.02	-	0.12 ± 0.01	0.12 ± 0.02	-	-	-	0.18 ± 0.02	0.18 ± 0.01	0.13 ± 0.008	0.10 ± 0.02	0.10 ± 0.01	0.18 ± 0.05
20	Methyl geranate	1323	1320.35	0.70 ± 0.10	0.33 ± 0.01	0.44 ± 0.08	0.47 ± 0.02	0.68 ± 0.02	0.56 ± 0.01	0.83 ± 0.05	0.74 ± 0.03	0.66 ± 0.05	0.44 ± 0.01	0.59 ± 0.02	0.02 ± 0.006
21	2-Oxabicyclo [2.2.2]octan-6-ol, 1,3,3-trimethyl-, acetate	1344	1337	0.23 ± 0.02	0.15 ± 0.02	0.14 ± 0.01	0.16 ± 0.01	0.14 ± 0.01	0.15 ± 0.01	0.11 ± 0.03	0.10 ± 0.009	0.17 ± 0.03	0.18 ± 0.02	0.08 ± 0.01	0.18 ± 0.03
22	*α*-Terpineol acetate	1352	1348.9	3.60 ± 0.38	2.62 ± 0.27	2.32 ± 0.22	2.60 ± 0.27	2.53 ± 0.07	2.59 ± 0.10	3.06 ± 0.26	2.65 ± 0.12	3.03 ± 0.13	2.60 ± 0.09	2.12 ± 0.10	3.08 ± 0.35
23	Geranyl acetate	1383	1377.27	4.59 ± 0.09	-	0.53 ± 0.01	0.48 ± 0.04	0.46 ± 0.02	0.49 ± 0.01	0.53 ± 0.05	0.48 ± 0.02	-	0.75 ± 0.06	0.61 ± 0.03	0.77 ± 0.03
24	Methyl eugenol	1405	1403.17	2.66 ± 0.23	1.33 ± 0.13	1.39 ± 0.13	1.64 ± 0.11	1.35 ± 0.04	1.27 ± 0.14	3.24 ± 0.14	3.06 ± 0.22	1.56 ± 0.08	1.66 ± 0.11	4.15 ± 0.16	0.54 ± 0.02
25	(*E*)-Caryophyllene	1415	1415.52	2.07 ± 0.16	1.55 ± 0.32	1.53 ± 0.08	1.34 ± 0.01	1.18 ± 0.03	1.36 ± 0.03	0.52 ± 0.06	0.70 ± 0.04	1.62 ± 0.17	0.36 ± 0.02	0.55 ± 0.02	0.20 ± 0.04
26	*α*-Humulene	1451	1449.53	1.26 ± 0.06	-	0.11 ± 0.01	0.09 ± 0016	0.14 ± 0.01	0.12 ± 0.01	0.11 ± 0.02	0.24 ± 0.01	0.10 ± 0.01	0.14 ± 0.01	0.14 ± 0.01	0.29 ± 0.04
27	Geranyl isobutyrate	1514	1512.08	-	-	0.24 ± 0.01	0.28 ± 0.01	0.28 ± 0.05	0.30 ± 0.02	0.20 ± 0.006	0.24 ± 0.02	-	0.29 ± 0.01	0.34 ± 0.02	0.30 ± 0.05
28	Caryophyllene oxide	1581	1573.03	0.14 ± 0.03	0.29 ± 0.02	0.37 ± 0.03	0.43 ± 0.03	0.51 ± 0.03	0.44 ± 0.04	0.52 ± 0.01	0.62 ± 0.01	0.42 ± 0.01	0.61 ± 0.04	0.47 ± 0.02	0.59 ± 0.05
29	*α*-Humulene epoxide	1606	1600.46	-	0.20 ± 0.004	0.27 ± 0.2	0.30 ± 0.001	0.32 ± 0.02	0.30 ± 0.02	0.29 ± 0.03	0.35 ± 0.03	0.28 ± 0.01	0.38 ± 02	0.33 ± 0.02	0.65 ± 0.05
Total		-		99.03	98.90	99.07	98.42	98.58	98.78	98.70	98.94	99.30	99.36	98.83	98.85

^a^ LIT RI: Relative retention indices taken from the Adams book. ^b^ KI: linear retention indices on DB-5 column, experimentally determined using homologue series of *n*-alkanes.

**Table 3:** Collection sites with abbreviations and geographical characteristics of myrtle population collected in Iran.

No.	Population Names	Abbreviation Name	Collection Site	Longitude	Latitude	Altitude (m)
1	Khoraman company	Khoraman	Khorramabad, Lorestan	4819′42″	3323′35″	1188
2	Sar-sarab	Sar-sarab	Sar-sarab, Khorramabad, Lorestan	4822′15″	3321′35″	1365
3	Padeghan Hamzeh	Padeghan	Padeghan Hamzeh, Khorramabad, Lorestan	4818′35″	3333′44″	1258
4	Poldokhter	Poldokhter	Cham-Mort, Poldakhter, Lorestan	4758′3″	3324′43″	905
5	Ilam	Ilam	Gavmishan Bridge, Darreh Shahr, Ilam	4732′4″	335′12″	564
6	Kermanshah	Kermansh	Sarab Mort, Gilane–Gharb, Kermanshah	4556′26″	347′44″	827
7	Kohgiluyeh–Boyer–Ahmad	Koh_BA	Kalat–Dehdasht, Kohgiluyeh–Boyer–Ahmad	5032′46″	3058′24″	726
8	Khuzestan	Khuz	Mongere, Andimeshk, Khuzestan	4811′23″	3258′24″	853
9	Kerman	Kerman	Dehsard, Kerman	5633′11″	2839′57″	1851
11	Yazd	Yazd	Pirnarestaneh, Yazd	322′20″	5430′18″	1701
10	Shiraz	Shiraz	Nurabad–Mamasani, Fars	5133′4″	309′10″	980
12	Hormozgan	Hormoz	Fareghan, Hormozgan	5615′10″	2757′40″	1463

## Data Availability

The data presented in this study are available on request from the corresponding author.

## Supplementary Materials

The following supporting information can be downloaded at: https://www.mdpi.com/article/10.3390/plants13243458/s1, Table S1: Correlation analysis between antioxidant activity, flavonoid, phenol, and essential oil content in *Myrtus communis* L.

## References

[1] Aleksic V., Knezevic P. (2014). Antimicrobial and antioxidative activity of extracts and essential oils of *Myrtus communis* L.. Microbiol. Res..

[2] Ghafouri F., Rahimmalek M. (2018). Genetic structure and variation in different Iranian myrtle (*Myrtus communis* L.) populations based on morphological, phytochemical and molecular markers. Ind. Crops Prod..

[3] Ghasemi Pirbalouti A., Craker L.E. (2015). Diversity in chemical compositions of essential oil of myrtle leaves from various natural habitats in south and southwest Iran. J. For. Res..

[4] Hennia A., Miguel M.G., Nemmiche S. (2018). Antioxidant activity of myrtus communis l. and myrtus nivellei batt. & trab. extracts: A brief review. Medicines.

[5] Giuliani C., Moretti R.M., Bottoni M., Santagostini L., Fico G., Montagnani Marelli M. (2023). The leaf essential oil of *Myrtus communis* subsp. tarentina (L.) Nyman: From Phytochemical Characterization to Cytotoxic and Antimigratory Activity in Human Prostate Cancer Cells. Plants.

[6] Matsehorova O.Y., Odyntsova V.M. (2024). Prospects for the creation of new phytochemical medicinal products based on *Myrtus communis* L. (a review). Curr. Issues Pharm. Med. Sci. Pract..

[7] Alipour G., Dashti S., Hosseinzadeh H. (2014). Review of pharmacological effects of *Myrtus communis* L. and its active constituents. Phytother. Res..

[8] Diaz A.M., Abeger A. (1987). *Myrtus communis*-chemical composition and biological activity of the extracted juice. A review. Fitoterapia.

[9] Mumivand H., Izadi Z., Amirizadeh F., Maggi F., Morshedloo M.R. (2023). Biochar amendment improves growth and the essential oil quality and quantity of peppermint (*Mentha*× *piperita* L.) grown under waste water and reduces environmental contamination from waste water disposal. J. Hazard. Mater..

[10] Shahbazian D., Karami A., Raouf Fard F., Eshghi S., Maggi F. (2022). Essential Oil Variability of Superior Myrtle (*Myrtus communis* L.) Accessions Grown under the Same Conditions. Plants.

[11] Mumivand H., Ebrahimi A., Shayganfar A., Khoshro H.H. (2021). Screening of tarragon accessions based on physiological and phytochemical responses under water deficit. Sci. Rep..

[12] Pank F., Kayser O., Quax J. (2007). Breeding of Medicinal Plants. Medicinal Plant Biotechnology. From Basic Research to Industrial Applications.

[13] Nurzyńska-Wierdak R. (2023). Chemical Diversity, Yield, and Quality of Aromatic Plants. Agronomy.

[14] Ramawat K.G., Arora J. (2021). Medicinal plants domestication, cultivation, improvement, and alternative technologies for the production of high value therapeutics: An overview. Medicinal Plants: Domestication, Biotechnology and Regional Importance.

[15] Messaoud C., Béjaoui A., Boussaid M. (2011). Fruit color, chemical and genetic diversity and structure of *Myrtus communis* L. var. italic Mill. morph populations. Biochem. Syst. Ecol..

[16] Hazrati S., Hosseini S.J., Ebadi M.-T., Nicola S. (2022). Evolution of phytochemical variation in Myrtle (*Myrtus communis* L.) organs during different phenological stages. Horticulturae.

[17] Cakir A. (2004). Essential oil and fatty acid composition of the fruits of *Hippophaer hamnoides* L. (Sea Buckthorn) and *Myrtus communis* L.. Biochem. Syst. Ecol..

[18] Flamini G., Pier Luigi C., Morelli I., Maccioni S., Baldini R. (2004). Phytochemical typologies in some populations of *Myrtus communis* L. on Caprione Promontory (East Liguria, Italy). Food Chem..

[19] Mele C., Corona L., Melito S., Raggi L., Mulas M. (2019). The genetic diversity of selections and wild populations of myrtle revealed by molecular geographic contexts. Ind. Crops Prod..

[20] Usai M., Marchetti M., Culeddu N., Mulas M. (2020). Chemotaxonomic evaluation by volatolomics analysis of fifty-two genotypes of *Myrtus communis* L.. Plants.

[21] Chryssavgi G., Vassiliki P., Athanasios M., Kibouris T., Michael K. (2008). Essential oil composition of *Pistacia lentiscus* L. and *Myrtus communis* L.: Evaluation of antioxidant capacity of methanolic extracts. Food Chem..

[22] Bajalan I., Pirbalouti A.G. (2014). Variation in antibacterial activity and chemical compositions of essential oil from different populations of myrtle. Ind. Crops Prod..

[23] Rahimmalek M., Mirzakhani M., Pirbalouti A.G. (2013). Essential oil variation among 21 wild myrtle (*Myrtus communis* L.) populations collected from different geographical regions in Iran. Ind. Crops Prod..

[24] Mumivand H., Khanizadeh P., Morshedloo M.R., Sierka E., Żuk-Gołaszewska K., Horaczek T., Kalaji H.M. (2021). Improvement of growth, yield, seed production and phytochemical properties of *Satureja khuzistanica* jamzad by foliar application of boron and zinc. Plants.

[25] Sunil C., Kumar V., Van Staden J. (2019). In vitro alpha-glucosidase inhibitory, total phenolic composition, antiradical and antioxidant potential of *Heteromorpha arborescens* (Spreng.) Cham. & Schltdl. leaf and bark extracts. S. Afr. J. Bot..

[26] Saffaryazdi A., Ganjeali A., Farhoosh R., Cheniany M. (2020). Variation in phenolic compounds, α-linolenic acid and linoleic acid contents and antioxidant activity of purslane (*Portulaca oleracea* L.) during phenological growth stages. Physiol. Mol. Biol. Plants.

[27] Jiang X., Liu Y., Li W., Zhao L., Meng F., Wang Y., Tan H., Yang H., Wei C., Wan X. (2013). Tissue-specific, development dependent phenolic compounds accumulation profile and gene expression pattern in tea plant [*Camellia sinensis*]. PLoS ONE.

[28] Li Y., Kong D., Fu Y., Sussman M.R., Wu H. (2020). The effect of developmental and environmental factors on secondary metabolites in medicinal plants. Plant Physiol. Biochem..

[29] Benlarbi K., Elmtili N., Macı F., Galindo J. (2014). Influence of in vitro growth conditions in the production of defence compounds in *Mentha pulegium* L.. Phytochem. Lett..

[30] Kabtni S., Sdouga D., Bettaib Rebey I., Save M., Trifi-Farah N., Fauconnier M.L., Marghali S. (2020). Influence of climate variation on phenolic composition and antioxidant capacity of *Medicago minima* populations. Sci. Rep..

[31] Mansinhos I., Gonçalves S., Romano A. (2024). How climate change-related abiotic factors affect the production of industrial valuable compounds in *Lamiaceae* plant species: A review. Front. Plant Sci..

[32] Medda S., Fadda A., Mulas M. (2022). Climate variables of the sites of origin and genotype influence on phenolic compounds accumulation in cultivars of *Myrtus communis* L.. Horticulturae.

[33] Mollaei S., Ebadi M., Hazrati S., Habibi B., Gholami F., Sourestani M.M. (2020). Essential oil variation and antioxidant capacity of *Mentha pulegium* populations and their relation to ecological factors. Biochem. Syst. Ecol..

[34] Spitaler R., Schlorhaufer P.D., Ellmerer E.P., Merfort I., Bortenschlager S., Stuppner H., Zidorn C. (2006). Altitudinal variation of secondary metabolite profiles in flowering heads of *Arnica montana* cv. ARBO. Phytochemistry.

[35] Yadav S.K. (2010). Cold stress tolerance mechanisms in plants. A review. Agron. Sustain. Dev..

[36] Khanizadeh P., Mumivand H., Morshedloo M.R., Maggi F. (2024). Application of Fe_2_O_3_ nanoparticles improves the growth, antioxidant power, flavonoid content, and essential oil yield and composition of Dracocephalum kotschyi Boiss. Front. Plant Sci..

[37] Jafari Khorsand G., Morshedloo M.R., Mumivand H., Emami Bistgani Z., Maggi F., Khademi A. (2022). Natural diversity in phenolic components and antioxidant properties of oregano (*Origanum vulgare* L.) accessions, grown under the same conditions. Sci. Rep..

[38] Dodd A.N., Jakobsen M.K., Baker A.J., Telezerow A., Hos S.W., Laplaze L., Barrot L., Poething R.S., Haselhoff J., Webb A.A.R. (2006). Time of day modulates low-temperature Ca^2+^ signals in *Arabidopsis*. Plant J..

[39] Beiranvandi M., Akbari N., Ahmadi A., Mumivand H., Nazarian F. (2022). Biochar and super absorbent polymer improved growth, yield, and phytochemical characteristics of *Satureja rechingeri* Jamzad in water-deficiency conditions. Ind. Crops Prod..

[40] Cheng L., Han M., Yang L.M., Yang L., Sun Z., Zhang T. (2018). Changes in the physiological characteristics and baicalin biosynthesis metabolism of *Scutellaria baicalensis* Georgi under drought stress. Ind. Crops Prod..

[41] Petretto G.L., Maldini M., Addis R., Chessa M., Foddai M., Rourke J.P., Pintore G. (2016). Variability of chemical composition and antioxidant activity of essential oils between *Myrtus communis* var. Leucocarpa DC and var. Melanocarpa DC. Food Chem..

[42] Mulas M., Melis R. (2011). Essential oil composition of myrtle (*Myrtus communis*) leaves. J. Herbs Spices Med. Plants.

[43] Wannes W.A., Marzouk B. (2016). Characterization of myrtle seed (*Myrtus communis* var. baetica) as a source of lipids, phenolics, and antioxidant activities. J. Food. Drug Anal..

[44] Mimica-Dukić N., Bugarin D., Grbović S., Mitić-Culafić D., Vuković-Gacić B., Orcić D., Jovin E., Couladis M. (2010). Essential oil of *Myrtus communis* L. as a potential antioxidant and antimutagenic agents. Molecules.

[45] Berka-Zougali B., Ferhat M.A., Hassani A., Chemat F., Allaf K.S. (2012). Comparative study of essential oils extracted from Algerian *Myrtus communis* L. leaves using microwaves and hydrodistillation. Int. J. Mol. Sci..

[46] Aghamirzaei H., Mumivand H., Nia A.E., Raji M.R., Maroyi A., Maggi F. (2024). Effects of Micronutrients on the Growth and Phytochemical Composition of Basil (*Ocimum basilicum* L.) in the Field and Greenhouse (Hydroponics and Soil Culture). Plants.

[47] Ahmadi S.Z., Zahedi B., Ghorbanpour M., Mumivand H. (2024). Comparative morpho-physiological and biochemical responses of Capsicum annuum L. plants to multi-walled carbon nanotubes, fullerene C60 and graphene nanoplatelets exposure under water deficit stress. BMC Plant Biol..

[48] Alkadi H. (2020). A review on free radicals and antioxidants. Infect. Disord. Drug Targets (Former. Curr. Drug Targets-Infect. Disord.).

[49] Mohammadian A., Karamian R., Mirza M., Sepahvand A. (2014). Effects of altitude and soil characteristics on essential of Thymus fallax Fisch. et CA Mey. in different habitats of Lorestan province. Iran. J. Med. Aromat. Plants Res..

[50] Yangui I., Younsi F., Ghali W., Boussaid M., Messaoud C. (2021). Phytochemicals, antioxidant and anti-proliferative activities of *Myrtus communis* L. genotypes from Tunisia. South Afr. J. Bot..

[51] Tuberoso C.I.G., Barra A., Angioni A., Sarritzu E., Pirisi F.M. (2006). Chemical composition of volatiles in Sardinian myrtle (*Myrtus communis* L.) alcoholic extracts and essential oils. J. Agric. Food Chem..

[52] Nooshkam A., Mumivand H., Hadian J., Alemardan A., Morshedloo M.R. (2017). Drug yield and essential oil and carvacrol contents of two species of Satureja (*S. khuzistanica* Jamzad and *S. rechingeri* Jamzad) cultivated in two different locations. J. Appl. Res. Med. Aromat. Plants.

[53] Mumivand H., Shayganfar A., Tsaniklidis G., Emami Bistgani Z., Fanourakis D., Nicola S. (2021). Pheno-morphological and essential oil composition responses to UVA radiation and protectants: A case study in three Thymus species. Horticulturae.

[54] Hamrick J.L., Godt N.J., Brown A.H.D., Clegg M.T., Kahler A.L., Weir B.S. (1989). Allozyme diversity in plant species. Plant Population Genetics; Breeding and Genetic, Resources.

[55] Shahbazian D., Karami A., Eshghi S., Maggi F. (2018). Variation in the essential oil yields and compositions of Myrtle (*Myrtus communis* L.) Populations collected from natural habitats of Southern Iran. J. Essent. Oil Res..

[56] Hendawy S.F., Khalid K.A. (2005). Response of sage (*Salvia officinalis* L.) plants to zinc application under different salinity levels. J. Appl. Sci. Res.

[57] Velioglu Y., Mazza G., Gao L., Oomah B.D. (1998). Antioxidant activity and total phenolics in selected fruits, vegetables, and grain products. J. Agric. Food Chem..

[58] Chang C.C., Ming-Hua Y., Hwei-Mei W., Jiing-Chuan C. (2002). Estimation of total flavonoid content in propolis by two complementary colorimetric methods. J. Food Drug Anal..

[59] Benzie I., Strain J. (1996). The ferric reducing ability of plasma (FRAP) as a measure of antioxidant power: The FRAP assay. Anal. Biochem..

[60] Jadidi M., Mumivand H., Nia A.E., Shayganfar A., Maggi F. (2023). UV-A and UV-B combined with photosynthetically active radiation change plant growth, antioxidant capacity and essential oil composition of *Pelargonium graveolens*. BMC Plant Biol..

[61] Adams R.P. (2017). Identification of Essential Oil Components by Gas Chromatography/Mass Spectrometry.

[62] Taheri-Garavand A., Mumivand H., Fatahi S., Nasiri A., Omid M. (2021). Modeling the kinetics of essential oil content and main constituents of mint (*Mentha aquatica* L.) leaves during thin-layer drying process using response surface methodology. J. Food Process. Preserv..

