# Supplementary material for: Natural Diversity in Total Phenol, Flavonoids, Antioxidant Properties, and Essential Oil Composition of Iranian Populations of Myrtus communis L
Source: Plants (Basel). 2024 Dec 10;13(24):3458. doi: 10.3390/plants13243458 (PMC11677132; doi:10.3390/plants13243458)
Supplement: Supplementary file 1 [file plants-13-03458-s001.zip › plants-3293369-supplementary.pdf]

Supplementary Table S1. Correlation analysis between antioxidant activity, flavonoid, phenol, and essential oil content in *Myrtus communis* L.

	FRAP (µg Fe/g DW)	Flavonoid (mg RE/g DW)	Phenol (mg GAE/g DW)	Essential oil content (mg/100 g DW)
FRAP (µg Fe/g DW)	1			
Flavonoid (mg RE/g DW)	0.93028**	1		
Phenol (mg GAE/g DW)	0.49064**	0.30862*	1	
Essential oil content (mg/100 g DW)	-0.61284**	-0.44622**	-0.90375**	1
